# Adapting a family intervention to reduce risk factors for sexual exploitation

**DOI:** 10.1186/s13034-020-00314-w

**Published:** 2020-02-18

**Authors:** Dawn T. Bounds, Caitlin H. Otwell, Adrian Melendez, Niranjan S. Karnik, Wrenetha A. Julion

**Affiliations:** 1 Department of Psychiatry and Behavioral Sciences, Section of Population Behavioral Health, College of Nursing, Community, Systems, & Mental Health Nursing, Rush University Medical Center, 1645 W. Jackson Blvd. Suite 600, Chicago, IL 60612 USA; 2 Department of Psychiatry and Behavioral Sciences, Section of Population Behavioral Health, Rush University Medical Center, 1645 W. Jackson Blvd. Suite 600, Chicago, IL 60612 USA; 3 Department of Psychiatry & Behavioral Sciences, Rush Medical College, Rush University, 1645 W. Jackson Blvd. Suite 600, Chicago, IL 60612 USA; 4 College of Nursing, Department of Women, Children and Family Nursing, Rush University Medical Center, 600 S. Paulina St. Suite 1080, Chicago, IL 60612 USA

**Keywords:** Homeless youth, Sexually exploited youth, Minority youth, LGBTQ+ youth, Family intervention, Focus groups

## Abstract

**Background:**

Sexually exploited youth are disconnected from societal tethers and need support systems, which makes them some of the most vulnerable of youth. This heightened level of vulnerability increases their risk for violence, abuse, ongoing sexual exploitation and all its sequelae. The purpose of this study was to examine an evidence-based intervention called STRIVE (support to reunite, involve and value each other) that has been a successful family re-engagement strategy with newly homeless youth. We sought to explore its contextual relevance for youth with risk factors for sexual exploitation and identify necessary adaptations to reduce risk factors for sexual exploitation. We deliberately took an intersectional approach in conducting this study.

**Methods:**

Six community-based focus groups were conducted with youth at risk for sexual exploitation and their service providers. Each group was recorded, transcribed, coded, and thematically analyzed.

**Results:**

Results from 29 youth and 11 providers indicate that there are unique considerations that must be taken into account while working with youth at risk for sexual exploitation to ensure effective service delivery and/or ethical research. Emergent themes included: *setting the stage* by building rapport and acknowledging experiences of structural violence, *protect and hold* which balances youth’s need for advocacy/support with their caregivers’ need for validation/understanding, and *walking the safety tightrope* by assessing risks and safety planning.

**Discussion:**

Focus groups are an effective methodology when working with traditionally disempowered populations particularly in gaining a range of perspectives to meet unique needs/preferences. Youth at risk for commercial sexual exploitation needs require strengths-based, individualized, multi-systemic approaches.

## Background

### Sexual exploitation

The key to preventing commercial sexual exploitation (CSE) in at risk youth may lie in bolstering family relationships and/or strengthening other supportive relationships. The Trafficking Victims Protection Act defines sex trafficking as sexual activity induced by force, fraud, or coercion or when a minor is induced to perform any sexual act regardless of the presence of force, fraud or coercion [1]. The Institute of Medicine/National Research Council (IOM/NRC) expanded on this definition by identifying commercial sexual exploitation and sex trafficking as a “range of crimes of a sexual nature committed against children and adolescents” that includes exploitation via prostitution, survival sex, pornography, sex tourism, early marriage, or performance in sexual venues (e.g. strip clubs).” [2]. Despite the existence of multiple definitions of CSE and sex trafficking in the literature, youth, particularly youth with socially complex needs are at risk. Youth with socially complex needs are youth who experience overlapping adversities such as the presence of Adverse Childhood Experiences (ACES), homelessness, and/or involvement in the child welfare or juvenile justice system.

#### Evidence-based interventions focused on sexually exploited youth

Moynihan et al. conducted a systematic review of interventions for youth who have been sexually exploited [3]. Very few interventions included social support (home-based/family involvement) as a component of their program. Interventions that specifically focused on health/social services did not target CSE, but rather, were designed to reduce the spread of disease. The residential programs aimed at supporting at-risk and recently sexually exploited youth addressed holistic concerns such as providing psychosocial, health, and vocational supports [3]. Of note, Moynihan highlighted the work of Robinson & Páramo (2007), Schram & Giovengo (1991), and Thomson, Hirshberg, Corbett, Valila, & Howley (2011), each of whom incorporated or discussed family involvement in their models [3]. The aforementioned review concludes by acknowledging the importance of employing practitioners or peer mentors who have experience with CSE and including cultural and family components into interventions moving forward.

Interventions specifically designed to prevent or directly address sexually exploited youth, are virtually absent from the research literature. Thus we also explored interventions for homeless youth [4]. In a recent systematic review, six evidence/based family re-engagement interventions were identified that addressed social support, service connection, or family functioning for homeless youth age 12 to 24 years old [4]. Of these, only one intervention (STRIVE; Support to Reunite, Involve, and Value Each other) included a component on reducing high-risk sexual behavior. STRIVE is a five session manualized intervention that is conducted with homeless youth and their parent or guardian. STRIVE sessions are intended to build upon family strengths, teach problem solving, conflict negotiation, and role clarification and is based upon cognitive-behavioral theories [5].

In order to address gaps in what we know about youth who are at risk for, or have experienced sexual exploitation, we conducted focus groups in an urban Midwestern city to understand the needs of youth at risk for sexual exploitation. Thus, the purpose of this study was to examine an evidence-based intervention that has been successful with newly homeless youth (STRIVE) to explore its contextual relevance for youth with risk factors for sexual exploitation.

## Theoretical framework

### Ecological model

In order to better understand CSE in the context of the lives of at risk youth, we used Bronfenbrenner’s Ecological model to map out the complex circumstances that may lead to CSE. Bronfenbrenner’s Ecological Model describes the social and cultural subsystems of the human–environment and the ways they interact and influence one another [6]. The ecological model presented by the IOM/NRC [2] poses four levels of risk factors (individual, relationship, community, and societal) that parallel Bronfenbrenner’s subsystems: microsystem, mesosystem, exosystem, and macrosystem. These four levels describe the circumstances and external conditions that may interact to make youth more susceptible to commercial sexual exploitation and trafficking.

#### Microsystem

The microsystem is a pattern of activities, social roles, and interpersonal relations that a developing person experiences in specific settings. The microsystem includes features that mediate the interactions in the immediate environment [6]. The individual risk factors include the youth’s own history that may expose them to other risk factors linked to exploitation. Adverse childhood experiences and ongoing adversity such as child abuse and neglect, homelessness (running away or being “thrown-away”), LGBTQ+ identity, and being involved with child welfare or criminal justice systems, all contribute to exposing youth to risky circumstances leading to their sexual exploitation [7]. Recent research confirms that youth who have been sexually exploited have the highest numbers of ACES, with sexual abuse being the most common [8, 9]. Poor family functioning or conflict is a significant contributing or exacerbating factor for running away or being asked to leave home [8–10].

Minorities, inclusive of sexual/gender minority youth are disproportionately overrepresented in current estimates of people who have been sexually exploited [8, 11, 12].

#### Mesosystem

The mesosystem links processes that occur between two or more settings in the youth’s life. For example, the interaction between parents and teachers reflects the ongoing communication and decision-making affecting a student at home and at school [6]. Relationship and community risk factors address the interactions with the people surrounding the youth, including family conflict, peer pressure, and social norms. Poor family functioning and family conflict can create a hostile home environment. Consequently, youth may try to either escape from the home environment or cope by engaging in risky behaviors. Risk factors are inclusive of individual and relationship risk factors. Combined levels of risk can lead to impacted psychological well-being and instability that push youth to look for support elsewhere [7]. Within the mesosystem, peer pressure and social norms may also encourage the youth to engage in risky behavior, including early consensual sexual activity, substance use, or gang involvement. This constellation of factors can magnify the risk of sexual exploitation [7].

#### Exosystem

The exosystem, which is similar to the mesosystem, is comprised of the linkages and processes that occur between two or more settings, but one setting is not directly encountered by the youth. Even so, events in the indirect setting influence the processes in the immediate setting. A common exosystem experienced by a child is the dynamic between home and the parent workplace [6]. Thus, parent’s employment and economic status and resulting stress directly impacts youth’s vulnerability to sexual exploitation as well as other adversity.

#### Macrosystem

The macrosystem is the societal blueprint for a particular culture or subculture. In the macrosystem, the micro- meso-, and exosystems share the same knowledge, belief systems, resources, and structures [6]. CSE exists within a “culture of tolerance,” [13] that is grounded in the oversexualization of youth, glorification of pimp culture, and objectification of women [14]. The normalization of buying sex on online forums also influences the demand for CSE [15]. Additionally, the broader society where youth live contributes to the difficulty combating CSE of minors. A central misconception regarding CSE is that it is primarily an international phenomenon. Contrary to this line of thinking, the majority of sex trafficking victims in the United States are U.S. citizens [16]. The general public has inadequate understanding of the prevalence of sex trafficking in the United States, the forces leading to a person being sex trafficked, and the characteristics of a person being sex trafficked [7]. During the course of their exploitation, it is likely that CSE may not be recognized. As many as 75% of CSE victims come into contact with health care professionals [17], but clinicians, nurses, and other staff interacting with victims are not trained properly or consistently on detecting possible signs of sex trafficking/sexual exploitation and therefore miss the opportunity to intervene [15].

### Intersectionality

Intersectionality is a theoretical framework that situates multiple microlevel experiences within macrolevel systems of privilege and oppression [18]. A limitation of most research and policy is the lack of acknowledgement that intersectionality exists in the lives of participants as well as our own lives [18]. Intersectionality acknowledges the complexity of inequity by considering the junctures of multiple social locations (e.g. race, gender, sexuality), power relations, and experiences [19]. Some intersecting vulnerabilities of youth in the current study include being a youth of color and/or LGBTQ+ and experiences such as homelessness, gender based violence, or having a history of trauma. The original STRIVE intervention will be adapted, through focus group methodology, for use in an urban Midwestern setting to reduce risk factors that predispose youth to sexual exploitation. We intentionally consider the unique needs of homeless/runaway, African American, and LGBTQ+ youth given the cumulative risk identified above for sexual exploitation. Taking an intersectional approach [20, 21] is a logical next step as we propose to take a proven intervention with homeless youth and refine the content and approach to take into account the layered risks associated with their age, race/ethnicity, sexual exploitation history, sexual/gender identity, and family functioning.

## Methods

### Design

The first author leveraged existing relationships in the community to recruit experts to participate in an expert focus group. Youth were recruited from the same community organizations as the content experts. The two initial focus groups were conducted with experts versed in youth homelessness, working with minority youth, and/or sexual exploitation in order to examine the STRIVE intervention for potential adaptations (n = 4; n = 7; Table 1). The experts included social service providers, social workers, counselors, and advanced practice nurses. Experts came from a variety of organizations including those that work with homeless and sexually exploited youth from settings such as drop-in centers, shelters, and school-based and community centers. The majority of the experts were female (n = 9) and from an ethnic/racial minority background (n = 6). Expert focus groups were conducted via video conference (WebEx). All experts were recruited through email and informed consent was signed electronically via REDCap. Experts were incentivized with ten dollar electronic gift cards. Next, youth focus group participants were recruited from youth-serving organizations (homeless shelters, school-based health centers, and community centers). Our content expert partners organized the focus group meetings and invited youth to participate in the study. Once the research team arrived, informed consent was completed prior to youth participation. Due to the sensitive nature of the discussion, demographic data were not collected from the youth. In order to protect student confidentiality, the only documents with youth names were the signed informed consent documents. Youth were also encouraged to refrain from using names in the focus group meetings. Four focus groups were held with youth (n = 29) whose ages ranged from 15 to 23 years old. One youth was White and the remaining 28 youth were African American. In order to maintain a safe environment, youth were not explicitly queried about sexual orientation and gender identity. Youth focus groups were held at four sites in the community. One group was held at a high school serving underserved youth during lunch time. A youth-serving community agency held a group for at-risk boys during their afterschool program. Groups were also held at one short-term and one long-term homeless shelter. Youth were incentivized with five dollar gift cards and provided with pizza and pop during the meeting. During the youth focus groups the STRIVE intervention was discussed for potential refinement.

**Table 1:** Expert Panelists

Expert panelist pseudonym	Content area expertise
Amy	LCSW who works with human trafficking survivors
Jaime	Case manager who works with human trafficking survivors and their children
Jasmine	Youth program manager who works with families of homicide victims
Kendra	LCSW who works with pregnant and parenting youth
Lisa	Director who works with LGBTQ+, homeless, and systems involved youth
Michael	LCPC who works with at-risk, LGBTQ+, and system involved youth
Nina	LSW who works with homeless youth
Rita	APRN who works with homeless and persistently mentally ill youth; consults on difficult mental health cases
Sam	LCSW who works with youth in school-based health and community health settings
Stephanie	Provides health equity education and training to providers in the community including systems of care (health and juvenile justice)
Veronica	APRN who works with LGBTQ+, system involved, and traumatized youth

### Procedures

This study was approved by the institution’s IRB. Each focus group followed the same procedure: introductions, informed consent process, and description of the STRIVE intervention, open-ended questions about considerations working with minority (ethnic/racial and/or sexual/gender) and/or marginalized youth (African Americans, homeless youth, sexually exploited youth, and/or LGBTQ+ youth). After allowing time to have an open discussion on population specific dynamics, we then proceeded to the interview guide which was primarily focused on the STRIVE intervention. The interview guide included the following questions: (1) what is missing from the described intervention, (2) what is the best way to recruit youth and parents/guardian/primary caregivers into the study, (3) how do we best talk to youth and their parents/guardian/primary caregivers about sensitive issues like sex risk-taking behavior, sexual exploitation, sexual/gender identity, (4) what adaptations need to be made to the language/terminology used in the intervention, and (5) what language/terminology should be used in the recruitment material?

The first author moderated the focus group discussion. Additional team members were present to take notes during the discussion, and attend to technical aspects of the research such as collecting signatures on consent forms and passing out incentives at the conclusion of the focus group. Every session was followed by a debriefing amongst the research team. The WebEx focus groups with content experts were video recorded; the groups lasted for 92 and 99 min respectively. Abridged transcripts were created from the videos and then uploaded into Dedoose Cross Platform Application [22] for analysis. Each youth focus group was digitally recorded. Youth groups lasted from 30 to 67 min. Digital recordings from the youth focus groups were transcribed verbatim, and uploaded into Dedoose for content analysis.

### Analysis

Content analysis was used to analyze the data from each focus group session by using systematic coding to identify trends, patterns, and discourses of communication [23, 24]. All focus group transcripts (content experts and youth) were independently coded by three different coders. Open coding was used, and each coder developed their coding frame as described in content analysis [24]. After independently coding all of the transcripts, the three coders met jointly over a series of 4 weeks to discuss similarities and differences in codes and to confirm emergent themes. Krefting asserts that trustworthiness in qualitative research is established by credibility, transferability, dependability, and confirmability [25]. Credibility was established by examining all transcripts and written notes, using the participants’ own words in quotes, and utilizing memoing during the coding process. Transferability was established by comparing narrative content across the various risk-related groups. Dependability and confirmability was ensured by adhering to consistent detailed analytical methods, building a unified coding frame, and using three independent coders.

## Results

Three themes were identified as a structure/process for engaging youth in a family intervention (STRIVE) who are at risk for sexual exploitation: (1) *Setting the stage*, (2) *Protect and hold*, and 3) *Walking the safety tightrope*. Each theme was further categorized as either adaptations to an engagement strategy to recruit and deliver STRIVE or adaptations to the content of STRIVE. Each theme represents a different system within the Ecological Model as well as phase in the project’s process: moving from the exo/macrosystem as we battle historical contexts during the *setting the stage* phase, to the mesosystem as we scaffold the family during the *protect and hold* phase, to finally the microsystem as we consider individual risks and needs of youth in the *walking the safety tightrope* phase.

### Setting the stage

*Setting the stage* refers to the initial phase of the relationship where building rapport and acknowledging experiences of structural violence are essential. *Setting the stage* is not a static concept, but rather an ongoing educational process that continues throughout the relationship in an effort to reduce stigma, express empathy, and manage misconceptions. The concept of building upon educational content as a means of establishing rapport, addressing misconceptions surrounding behavioral health, and validating experiences fits well within the existing structure of STRIVE as a psychoeducational intervention.

Being relatable was emphasized by both experts and youth across settings. One youth articulated:

You gotta relate to what people are goin’ through. You gotta be able to, I guess, like if you’re not from the hood you gotta be able to put on the shoes of somebody that is. You gotta try to see it from somebody else point of view regardless of what you’re comin’ from.

Similarly, Nina from the expert panel highlighted that trusted adults may be reticent to participate in the intervention because of concerns about maintaining privacy, “Black people don’t want people coming in and getting into their business.”

An additional group of experts expressed their perceptions about the potential challenges associated with engaging African American youth in the STRIVE intervention:

**Veronica:** I think you need therapists that are African American. I mean I think that would go over better with families. Again it decreases the stigma that they are working with someone who is African American and is in a healing profession. It would decrease some of the worries of being involved in a research study and distrust of researchers in general.

In particular several content experts expressed that families may be concerned about the potential for becoming involved in child protective services. Veronica went on to say, “Some families are going to be scared that DCFS is going to get involved.” Both youth and content experts stressed the importance of considering the historical contexts of research in the African American community which is magnified when the focus is on the topic of mental health and wellbeing. Recommendations for working with sensitive topics such as mental and sexual health require a cultural lens that reflects an understanding of the population being served.

A second emergent consideration during the *setting the stage* phase was the high prevalence of misconceptions and alternative perspectives surrounding sexual exploitation. This is aligned with existing data that suggests that many victims of sexual exploitation do not identify as victims, nor do they label their relationships as exploitative [8]. Content experts in the current study emphasized these misconceptions. Veronica noted the following: “you don’t want to marginalize them or turn them off to the study by initially declaring them exploited cause that’s not how they see themselves necessarily. Some will– some won’t.” Amy, a content expert colleague concurred by voicing the following perspective, “Some people will never identify as experiencing exploitation. And so changing that language would be helpful to get people engaged.”

Finally with regard to the importance of addressing misperceptions around the concept of sexual exploitation, another content expert, Jasmine voiced the following perspective:

I would like to add a piece of psychoeducation in there. In regards to sexual exploitation—what does exploitation look like? Because sometimes I find that they use different language for what it is so that it becomes acceptable for them to actually live in that kind of environment and not feel like, you know, they’re being judged or as if they’ve made a bad choice…not assuming that they recognize that this relationship is defined as something that is exploiting them sexually.

In accord with this line of thinking from the experts, youth often did not perceive trading sex or engaging in unwanted sexual behavior as unsafe or exploitative, even though they may have been homeless and trading sex for survival. Content expert, Michael stressed the need to discuss how to get basic needs met in a healthy way with youth because:

Whatever they might be doing to get their needs met in some way might be unhealthy. Right…Just being able to have a discussion about getting those basic needs in a healthy way… Half the time they’re not aware that getting those needs don’t have costs to it.

The following perspective from a youth focus group participant emphasized the need for education on the concepts of sexual exploitation and healthy relationships:

I never once had anybody take advantage of me. I’m not gonna let you do something I don’t want you to do. I’m not gonna let you. As far as in a sexual matter. I ain’t gonna lie. People just don’t care [crosstalk] People just gonna do what they wanna do. It’s like you can bring a horse to water, can’t make ‘em drink you know what I mean. You can’t take a person out of a situation. My momma still on drugs. I can’t send her to rehab if she don’t wanna do it.

### Protect and hold

The process of *protect and hold* illustrates an ongoing process in which the researcher/interventionist balances the youth’s need for advocacy/support with their caregivers’ need for validation and understanding. This balance occurs within a family system potentially marred by ongoing conflict. Concerns about family conflict permeated each focus group discussion. One content expert provided the following insight related to this theme, “You can hold the parent, but you have to 100% back the youth.”

Another concern voiced by experts was the lack of support these youth currently have for a variety of reasons that can be seen in the following dialogue:

**Veronica**: There is a whole population of kids that don’t have anybody right…they don’t know many adults you know and I feel they don’t trust adults for good reason.

**Nina**: You’re going to really have to incentivize the hell out of this thing because it’s going to take an act of God to get these trusted adults to invest in this process… unfortunately the majority of young people that I work with just don’t seem to have adults in their lives who are invested in them like this.

Advocating for youth during potentially malfunctioning family dynamics is a child-centered approach that has the potential to build youth self-worth and model alternative pathways to resolve familial disagreement. It can reinforce the adult role as protector while simultaneously validating their perspective and feelings. This approach has the potential to build empathy amongst family members as well as model alternative strategies to resolve conflict that defuses the anger and resentment that can contribute to youth tendencies to run away… This group of content experts highlighted the challenges inherent with working with marginalized youth and their families:

**Sam**: It seems like the original assumption is that the family or the adults in the family, the parents, want to reunite with the child. And what I’ve seen is while that’s certainly valid I have seen families where either the mother was so distracted by other things happening in her life or her current partner/boyfriend that she just never had any real time to deal with the child. So the child leaving the household was almost welcomed by the mother. Because she no longer had to deal with that responsibility; she was really pursuing her own life rather than acting more as a parent.

**Jasmine**: I would also like to add that sometimes the caregiver/parent has some real fears around the child being a presence in the home because there may be a connection to recurring violence or there may be some untreated symptoms… There is this fear factor with reuniting with the runaway child because they’re not sure that they’re not the cause of some of the trauma that’s coming in the home or the violence in the community that they’re surrounded by.

The following experts highlight the need to balance family needs. They felt the need to advocate for youth and simultaneously validate the parents’ concerns within the context of a family intervention that focuses on negotiation and problem solving. Balancing family needs by advocating for youth and validating the parents’ concerns within the context of a family intervention that focuses on negotiation and problem solving was highlighted in the following dialogue amongst experts:

**Sam**: Well I think one of things for a lot of youth that I see is they just need a consistent advocate in their lives. And what I mean by that is for a lot of kids that are living on the margins…things like advocacy things that we take for granted, they can’t… I’ve seen countless kids who they left their family of origin, their immediate nuclear family, but now that they’re homeless; they have no relatives that they can even go crash on the couch with. The family itself is sort of splintered and so things like going to sleep on your cousins couch for a couple of nights while things cool off in that house, that’s not really an option for them because their cousins or their aunts, their grandmothers, just don’t feel enough of a kinship to them to allow those sorts of things.

**Jasmine**: I would also like to suggest, or what has worked for us is to support the reality of the fears that the caregivers or that supportive participant has. The caregiver or whoever that youth has chosen as the person who’s willing to walk alongside them for the sessions. You know up front identifying and kind of affirming those are real fears but then also saying how can we make this environment better or how can we support the goal which is to kind of offer skills or coping mechanisms for this youth to be able to re-engage back in the family.

Later Sam emphasized the need for:

Family reconciliation services. If the youth may have done something that may have offended someone in the family or if the family did something that may have offended the youth. Sometimes there needs to be somebody that can get both parties back into the same room to sort of reconcile…sometimes it just needs to be, I don’t want to say refereed but sort of a moderator so that two people can reconcile and get the youth back into the household because at some level it’s just egos, competing egos that are preventing the family from reengaging.

The youth also reinforced how family conflict was central to their homelessness by expressing:

I don’t know, ‘cause a lot of people who run away, they just can’t go back home after they run away, ‘cause they feel like they might get in trouble.

### Walking the safety tightrope

*Walking the safety tightrope* refers to the process of ongoing risk assessments and safety planning. Due to homeless youths’ propensity to engage in high risk behavior, safety is of paramount importance. It is important to not assume that a psychoeducational program such as STRIVE will mitigate these risks immediately. Therefore, ongoing risk assessments and safety planning are vital.

The need to get to know youth and their personal experiences was coupled with the need to ensure safety. This theme was endorsed by both youth and content experts.

I think that a lot of people (need to) tell their stories first…You all know we homeless so that’s why we’re here, but you don’t know our stories…like let me tell you a story first. Then, after they tell their story, then you can say, all right, so this is how STRIVE can benefit you.

Experts echoed a similar concern since the STRIVE intervention is intended for youth who may leave home without permission or be asked to leave due to conflict. Lisa emphasized the following:

I would also say that some conversations around like harm reduction (are needed). If young people are already removed from their homes or have already runaway. What I think is important to do is some information gathering around what are the other safe places they identified or flee to. You know what we observe is (that) often the environment (they flee to) may be safer in one context…whatever was the factor that was so bad that the young person left, they’re not experiencing that in the new place that they’re at but there might be a whole other crop of risk factors that they’re exposed to in that new environment.

A content-related theme that emerged from both the experts and youth while *walking the safety tightrope* was related to the need for resources related to the youth’s survival (i.e. transportation, securing employment, or housing). Experts stressed the need to be prepared to navigate the multiplicity and complexity of needs inherent in working with vulnerable youth. Stephanie stressed the following:

Because of how intense this target population that you are looking to work with (is), are you considering other organizations that are working with this target population that can be that added support? Because, you know, I worry if you’re just a standalone in this, what is the likelihood of the commitment and the involvement students would have in participating in this study?.

Youth reinforced how we must be ready to assist in meeting their survival needs, as highlighted in the dialogue below:

**Interviewer**: Let’s talk a little bit about young people who had to trade sex to survive. You end up sleepin’ with somebody ‘cause you needed a place to stay, you needed somethin’ to eat, you needed a ride, whatever the case might be. Like I said, this could be somebody that you know that has experienced this. Could STRIVE help that person?

**Youth 1:** Could it? If I was trading sex for a ride, would you be able to give me a free ride? If the answer is yes, then sure. If the answer is no, then I doubt it.

In addition to being prepared to meet their needs, expert panelist, Lisa poignantly reminded us of the need for a non-judgmental approach by stating,

If we are not going to give them money then we need to be really careful about how we criticize the way they are getting money because it doesn’t mean they’re gonna stop getting money that way, it just means that they’re going to stop talking to us about it.

She also encouraged that we consider how service environments can also create tension for youth when she stated that:

With armed security or police there’s an adversarial relationship between many members of the African American community and the police. So what ways might we set up young people to already be tense or uncomfortable or feel criminalized as they’re coming into services for the intervention…Having an affirming or empowering environment as opposed to one where young people are initially criminalized and their behavior is being surveilled… (or) reacted to with force. I think removing that dynamic may also allow young people to feel more comfortable and express themselves.

## Discussion

The ecological systems framework is grounded in intersectionality, and provides a solid context for examining commercial sexual exploitation (CSE) in youth. What we know about CSE has evolved over time and spans from the micro-to macrosystem of the ecological model. Initially, microsystem research on CSE explored the lived experiences of youth impacted by CSE [26–28]. Youth homelessness, which crosses multiple systems, ranges from the micro to the meso level because of the multiple factors that are influential. For example, youth behaviors at the micro-level can be directly related to CSE, and family and economic contexts at the meso level can be contributory. Even so, we are also starkly aware that ACES, as well as ongoing adversity, can contribute to housing insecurity [29]. Since CSE is a common risk for homeless youth, the paucity of data on the effects of CSE on the mental health of African American and sexual/gender minority children and adolescents reflects a significant scientific gap [30]. Our initial premise was that there was likely to be an overrepresentation of youth with diverse social identities (minority status, sexual orientation, gender identity) in homeless populations. Therefore, we chose a validated family re-engagement intervention (STRIVE) for refinement to better address cultural and social concerns specific to youth risks for sexual exploitation. The experts and youth in our study endorsed the need for a youth-centered intervention approach.

Sahl and Knoepke note that youth who have been victimized by CSE often encounter multiple systems (i.e. juvenile justice, child welfare, community mental health). These systems tend to be prescriptive in their approach and fail to consider the youth’s voice [31]. When youth voices are not considered in systems of care, there is a greater likelihood that youth will not adhere to treatment, run away and be mistrustful of providers. Youth who typically respond to family stress and discord by running away may be more likely to engage in an intervention that includes them as equal partners in the assessment and safety planning process from the onset. Our findings mirror the need to include the youth voice throughout the engagement process.

Macrosystem level research has concentrated on public policy to examine the extent of CSE and evaluate the need for services to help individuals impacted by CSE [7, 32]. Confusion about sexual exploitation, particularly indicators and definitions of sexual exploitation, has constrained the field [33, 34]. In Pearce’s exploration of sexual exploitation with practitioners in the field, she noted that lack of clarity and confusion amongst both practitioners and youth can contribute to missed and dismissed legal cases [35]. Similarly when parents have difficulty identifying victimization, opportunities for intervening will be diminished.

It is well-documented that victims of sexual exploitation often do not self-identify as victims [7] and report “choosing” to enter the sex trade. Due to the traumatic nature of exploitation, youth may have even greater difficulty than adults disclosing that they are engaging in commercial sex trade as a survival mechanism [35]. Nevertheless, since federal law asserts that minors cannot freely choose to engage in commercial sex, they are therefore viewed as victims of sex trafficking [1]. Despite debate about youth-agency, practitioners in Pearce’s study agreed that a child-centered approach that puts the needs and best interest of the child at the forefront is best practice [36]. Thus the practitioner’s role should encompass empathizing with the youth’s experience, guiding them past self-blame, and facilitating relationships that aim to provide comfort, closeness, reassurance, reductions in anxiety, and promote exploration, learning, and psychosocial development [36, 37].

Of late, researchers have been more attentive to examining CSE in groups perceived to be at greater risk or who had historically been omitted from prior research [38]. This exploration of risk includes a multisystem and intersectional framework. Findings indicate that a few adaptations to the STRIVE intervention (see [5] for a summary of STRIVE sessions) [5] are necessary prior to engaging with youth with socially complex needs, such as being at high risk for sexual exploitation. Adaptations to both the content and delivery of STRIVE are needed to *set the stage* to both build an effective therapeutic alliance (delivery adaptation), as well as engage in an ongoing educational process in order to challenge misconceptions around sexual exploitation (content adaptation). In addressing the need for rapport building and intentional acknowledgement of structural violence, we make several recommendations:

(1) Establishing a youth advisory board for ongoing consultation, partnership, and accountability. In order to insure that youth voices are heard, we intend to create space for youth involvement by developing a youth advisory board. Community advisory boards are foundational to community-based participatory research [39]. Our findings suggest the need to have a continued connection with the youth we serve to ensure transparency, relatability, and cultural humility. Cyril et al. identified how mutually beneficial relationships and the bi-directional learning that occurs with community advisory boards can improve the health of vulnerable populations [40]. The iterative process of engaging with community advisory boards is not only valuable to vulnerable populations, it is also beneficial to the members of the clinical research team. Community engagement on this level has been identified as not only a way to amplify voices in marginalized populations but also a way to combat health disparities [41]. Fisher and Mustanski argue for inclusion of sexual minority youth as stakeholders during the development and implementation of responsible research as a moral imperative [42].

(2) Intentionally prioritizing ongoing training among the members of the research team on cultural humility. This recommendation addresses the need for transparency and relatability of researchers and clinicians, which can contribute to increased engagement. In particular, we must connect with youth in ways that resonate with the contexts of their lives. We also acknowledge the need to address ongoing education related to sexual exploitation with youth. This education should include, “person-centered” and “body/self-positive” sex education that includes an open, clear educational component on sex and healthy relationships. This particular content needs to be added to the curriculum for both youth and parents. Additionally, findings from focus groups in the current study reveal that family conflict remains a primary reason that youth leave the home and engage in high-risk behaviors. Thus, it is vital that high therapeutic emphasis should be focused on family functioning in this population. We assert that no further adaptations to the STRIVE intervention are necessary to *protect and hold* the family at this time. Even so, content and delivery adaptations to effectively *walk the safety tightrope* with youth who are at a high risk of sexual exploitation are indicated. Specifically it will be important to incorporate separate individualized assessments for the youth and their family member that focus on safety planning (engagement), as well as education and community referrals for life-skills training and addressing immediate needs (content). These findings indicate that until safety is assured, ethical considerations preclude a clinician from focusing on family functioning - and until the youth’s immediate needs are met, treatment progress will be precarious at best. A clinician should be prepared with resources and referrals that not only include ways youth can get their basic needs met but also how to navigate services and systems that help them secure jobs and ultimately survive.

(3) Getting needs met was central for the youth who participated in our study. Melrose notes that entry into the commercial sex trade is often predicated around the concept of meeting unmet financial and employment needs [43]. We would be remiss to ignore the social and economic context of risks associated with homelessness and sexual exploitation in our prevention efforts. To address this need, we plan to create a resource and referral list that would assist in addressing survival needs as they arise. We also see STRIVE as a potential entryway to other mental health services such as family therapy and therefore we will need to be prepared to facilitate ongoing family-focused treatment.

### Implications

In order to prevent sexual exploitation and disrupt the trajectory of runaway and homeless youth, intervention adaptation of existing efficacious interventions is warranted. As one of the youth focus group participants put it, “When you see something for so long, you gonna start doing that ‘cause you’re not gonna know another way.” Although it is difficult to meet all of the needs of vulnerable youth within the context of a research study, future efforts should explore the impact of embedding family reunification research into existing interventions within the community to ensure the availability of safety nets for youth. Additionally, relationships matter, and future research must also consider how these relationships—whether they include a family member, trusted adult, or provider—impact the trajectory of vulnerable homeless youth. Chisolm-Straker et al. along with other literature on at risk youth reinforces the need to focus on families [44]. In fact, one study found that the main difference between homeless youth who were trafficked and those who were not trafficked was the presence of a supportive adult. STRIVE’s primary focus is on improving family functioning via the development of communication and problem solving skills. Re-engaging youth with their family and/or a trusted adult is essential to their development [45]and also potentially protective against sex trafficking [44].

### Limitations

There are a few limitations that should be considered while interpreting our findings. First, we used co-ed groups for the majority of the focus groups. It is possible that some youth did not feel comfortable in this setting and therefore may not have freely expressed themselves; their responses may have been influenced by social desirability. We also did not hold focus groups that were explicitly comprised of lesbian, gay, bisexual or transgender specific groups. Given the stigma still associated with being a sexual and/or gender minority, this limitation may have influenced a comprehensive exploration of the needs of these specific youth. This might mean that we have not captured all of their needs, particularly with regard to family conflict and survival needs. Since 40% of homeless youth belong to the LGBTQ+ community [46], we will have to consider their needs on an individual level at intake into our program.

One of the youth focus groups was an all-male group, and in general, there were more boys than girls who participated in our youth focus groups. Given the fact that girls have historically been more impacted by CSE, this inequity may have impacted our findings. Lastly, we chose to focus on youth at risk for sexual exploitation rather than those who have previously been exploited. Because we did not ask, we do not know which members of the youth focus groups have a history of exploitation. It is very possible that the needs of youth at risk for CSE and those who have experienced CSE differ given the psychological trauma inherent in specific types of sexual exploitation, such as forced sex trafficking. Furthermore, the documented confusion in nomenclature in the field of sexual exploitation suggests that we cannot assume that there was universal understanding and/or agreement of the concept of CSE amongst the focus group participants. Despite these limitations and the tension between lack of youth voice and acknowledged agency of youth who trade sex, the findings from the focus groups remain relevant across several contexts.

## Conclusion

In conclusion, adapting targeted prevention strategies for at-risk youth may hold promise for addressing complex phenomena such as sexual exploitation. The recurrent theme of family conflict in our focus groups reinforced the need for a family based intervention such as STRIVE. Existing family focused, evidence-based interventions have great potential to be adapted and implemented with at risk youth in the fight against commercial sexual exploitation. Ultimately, this fight must be inclusive of intersectional/ecological frameworks that situate this body of work within multiple and complex systems that impact the lives of youth and their families.

## Data Availability

All data generated or analyzed during this study are included in this published article.

## Abbreviations

IOM: Institute of Medicine
NRC: National Research Council
ACES: Adverse childhood experiences
LGBT: Lesbian, gay, bisexual, transgender
STRIVE: Support to Reunite, Involve, and Value Each Other
CSE: Commercial sexual exploitation
